# Three-dimensional printing of polycaprolactone/hydroxyapatite bone tissue engineering scaffolds mechanical properties and biological behavior

**DOI:** 10.1007/s10856-022-06653-8

**Published:** 2022-03-10

**Authors:** Naghme Rezania, Mitra Asadi-Eydivand, Nabiollah Abolfathi, Shahin Bonakdar, Morteza Mehrjoo, Mehran Solati-Hashjin

**Affiliations:** 1 Faculty of Pharmacy, University of Montreal, Montreal, Canada; 2 Department of Biomedical Engineering, Amirkabir University of Technology, Tehran, Iran; 3 ZistnegarAmirkabirLtd, Hafez Ave, Tehran, 1591639802 Iran; 4 Iran National Cell Bank, Pasteur Institute of Iran, Tehran, Iran

## Abstract

Controlled pore size and desirable internal architecture of bone scaffolds play a significant role in bone regeneration efficiency. In addition to choosing appropriate materials, the manufacturing method is another significant factor in fabricating the ideal scaffold. In this study, scaffolds were designed and fabricated by the fused filament fabrication (FFF) technique. Polycaprolactone (PCL) and composites films with various percentages of hydroxyapatite (HA) (up to 20%wt) were used to fabricate filaments. The influence of (HA) addition on the mechanical properties of filaments and scaffolds was investigated. in vitro biological evaluation was examined as well as the apatite formation in simulated body fluid (SBF). The addition of HA particles increased the compressive strength and Young’s modulus of filaments and consequently the scaffolds. Compared to PCL, Young’s modulus of PCL/HA20% filament and three-dimensional (3D) printed scaffold has increased by 30% and 50%, respectively. Also, Young’s modulus for all scaffolds was in the range of 30–70 MPa, which is appropriate to use in spongy bone. Besides, the MTT assay was utilized to evaluate cell viability on the scaffolds. All the samples had qualified cytocompatibility, and it would be anticipated that addition of HA particles raise the biocompatibility in vivo. Alkaline phosphatase (ALP) evaluation shows that the addition of HA caused higher ALP activity in the PCL/HA scaffolds than PCL. Furthermore, calcium deposition in the PCL/HA specimens is higher than control. In conclusion, the addition of HA particles into the PCL matrix, as well as utilizing an inexpensive commercial FFF device, lead to the fabrication of scaffolds with proper mechanical and biological properties for bone tissue engineering applications.

**Figure:**
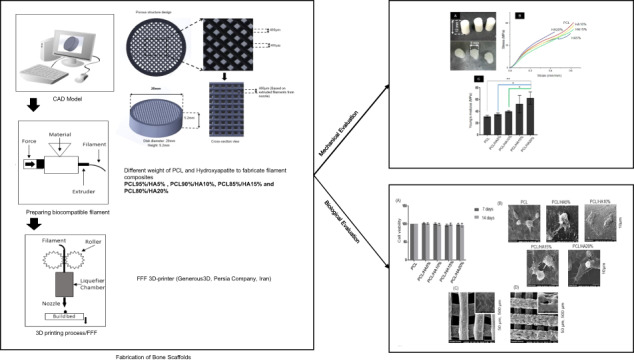
Graphical abstract

Graphical abstract

## Introduction

Bone is a porous composite structure consisting of hydroxyapatite (HA) crystals in a polymeric matrix [1] that is damaged owing to multiple reasons such as fractures, cancer, traumas, and osteoporosis. Autografts, allografts, and xenografts are the most common therapeutics with the limitation of disease transfer, induce immune system response, insufficient biocompatibility, and lack of donor sites [2, 3].

**Fig. 1:**
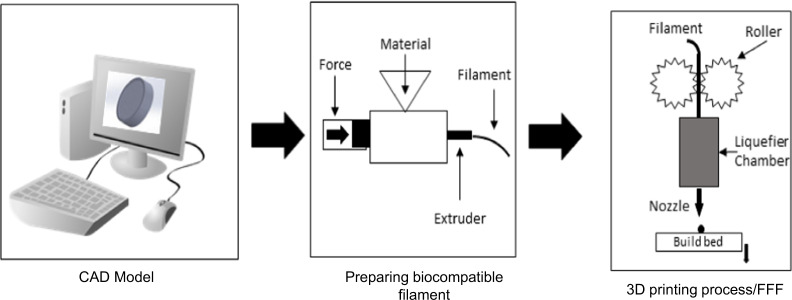
The schema of the fused filament fabrication (FFF) process, from left to right, shows the computational design of scaffold, filament fabrication, and 3D printing process of scaffolds

Nowadays, bone tissue engineering is an alternative way to repair bone failures. The main goal of tissue engineering is to fabricate a three-dimensional (3D) porous structure that mimics the extracellular matrix (ECM) and supports mechanical and biological properties, named scaffold. The bone scaffold should be biocompatible, biodegradable, and have sufficient mechanical strength, porosity, and interconnectivity for bone applications [2, 4–9]. Although traditional methods can produce highly porous scaffolds, they have a limitation on internal scaffold design and fabrication of reproducible interconnected porous structures for cell growth, migration, diffusion of oxygen, and waste removal [4–6, 9]. Rapid Prototyping (RP) techniques have provided superior progress in manufacturing predesigned tiny porous structures with an arbitrary internal structure that is a promising method to fabricate scaffolds with appropriate mechanical and biological properties for bone tissue engineering applications [10, 11]. Indeed, these methods can produce objects layer-by-layer according to the 3D design [10, 12, 13]. Fused filament fabrication (FFF) is an inexpensive RP technique that has been widely used for fabricating desirable scaffolds by controlling the internal and external structures for tissue engineering [5, 9, 11]. In the FFF process, as shown in Fig. 1, the filament is melted in a liquefier chamber and deposited layer-by-layer through a nozzle to fabricate a 3D sample straight from a computer-aided design (CAD) model. After each step, the build bed comes down, and the next layer is deposited on the previous layer until the predesigned object is completed [4, 10, 12].

In addition to the significant role of the manufacturing method for the fabrication of successful bone scaffold, the selection of appropriate material (biocompatible, biodegradable, and bioactive) is also considerable. A wide range of polymers, natural (Collagen, chitosan, hyaluronic acid, etc.) and synthetic (PLA, PGA, and PLGA), are utilized in bone tissue engineering. Although natural polymers have excellent properties to enhance bone regeneration, their drawbacks, such as immunogenetic response, weak mechanical properties, etc., limit their applications in bone tissue engineering. Therefore, using synthetic polymers is an alternative way to cure bone defects. Synthetic polymers have some advantages, including tailored mechanical properties, reproductivity, and more durability. Moreover, their good nature, such as temperature resistance properties, makes them appropriate for fused filament fabrication (FFF) [1, 14–16].

In this project, Polycaprolactone (PCL) and HA were chosen as scaffold’s material. The supreme features of (PCL), including biocompatibility, long–term biodegradability, excellent mechanical properties, and FDA approval, make it an appropriate material for bone tissue engineering applications. Besides, it is also printable with the FFF device [6, 11, 17–21]. Hydroxyapatite is a biocompatible and bioactive mineral structure similar to the natural bone widely used as a bone substitute. Recently, polymer/ceramic compositions such as PCL/HA have been utilized to generate bone scaffold that enhances cell proliferation and bone regeneration in vivo [2, 5, 11]. Therefore, selecting a suitable material and fabrication method result in a scaffold with sufficient structure and properties.

The previous studies dealt with the fabrication of 3D-printed composite bone scaffolds. Although they showed significant results, the lack of necessary experiments is evident in their works. Kim et al. (2017) evaluated the effect of HA percentage (0, 10, 15, and 20%) on the mechanical and biological properties of scaffolds fabricated by the 3D-plotting system and showed this process resulted in highly porous scaffolds (porosity 78%). They showed that the addition of HA particles had enhanced the mechanical properties (Tensile and compression strength) and good bioactivity properties. Despite the FFF method, which is based on filament melting and extrusion, the 3D-plotting system applies air pressure for material extrusion [11]. Bruyas et al. (2018) investigated the effect of composition (0–60% β-TCP/PCL) on the properties of scaffolds fabricated by FFF. They showed that increasing β-TCP had improved the mechanical, biological, degradation, and surface properties as a bone scaffold. However, they did not examine the bioactivity of specimens and rheology behavior during printing [17]. Using the extrusion-based additive manufacturing system, Huang et al. (2018) fabricated a PCL-based scaffold with 10% and 20% (HA and β-TCP). The results showed that scaffolds containing HA had better biological properties and lower mechanical properties than the scaffolds encompassing TCP. However, the addition of these two ceramic particles did not affect the wettability [22]. Maria et al. (2021) reported the 3D-printed PCL/HA (70/30%wt) scaffolds for bone tissue engineering applications. They prepared the mixture of PCL/HA powder and used Laser Powder Bed Fusion (LPBF) technology to fabricate these scaffolds. They evaluated scaffolds’ morphological properties, porosity, mechanical and biological behavior [23].

In this study, predesigned porous bone scaffolds (pore size of 400 µm and porosity of 37% to enhance bone regeneration) were fabricated by the commercial and low-cost FFF device. First, HA was synthesized, then profitable composite films were prepared with different percentages of HA (0%wt, 5%wt, 10%wt, 15%wt, and 20%wt) to support mechanical and biological properties compared to the bone structure components. The range of HA percentage was chosen based on the rheology and printability of the filaments. After filaments fabrication with the prepared composite films, the scaffolds were printed. The effect of various percentages of HA on the mechanical and biological properties of the scaffold was examined. The scaffolds showed appropriate mechanical properties compared to some previous studies. Besides, their bioactivity and biological properties were desirable. Hence, this research could be a road map for those who desire to fabricate the lowest price bone scaffolds with the aid of 3D printing techniques. Utilizing an inexpensive device for the fabrication of scaffolds helps to decrease the final price of the sample, which is a very important factor for developing medical tools and making them affordable for everyone. It is beneficial for human beings. Therefore, these 3D-printed PCL/HA scaffolds are promising for bone tissue engineering applications.

## Material and methods

Polycaprolactone (MW = 80,000 and Sigma-Aldrich, Germany) in the form of granules, Dimethylformamide (DMF, Sigma Aldrich, Germany), and synthesized hydroxyapatite powder were used.

### Synthesize of hydroxyapatite powder and characterization

Hydroxyapatite powder was synthesized by the chemical precipitation method [24]. First, 0.15 M calcium nitrate 4-hydrate solution [Ca(NO_3_)_2_.4H_2_O, 98%, Merck, Germany], and 0.09 M diammonium hydrogen phosphate solution [(NH_4_)_2_HPO_4_, 99%, Merck, Germany] were prepared. Afterward, the addition of 1 M sodium hydroxide solution [NaOH, 99%, Merck, Germany] brought the pH of the two solutions to about 11. The precipitation of HA was resulted from adding phosphate solution dropwise into a calcium nitrate solution. The following lines show reactions. The X-Ray Diffraction (Equinox model, France) with Cu–Kα radiation was employed to identify the crystalline structure of HA powder (JCPDS files were used).

1 $$10{{{\mathrm{Ca}}}}^{ + 2} + 6{{{\mathrm{HPO}}}}_4^ - + 2{{{\mathrm{OH}}}}^ - \to {{{\mathrm{Ca}}}}_{10}\left( {{{{\mathrm{PO4}}}}} \right)_6\left( {{{{\mathrm{OH}}}}} \right)_2 + 6{{{\mathrm{H}}}}^ +$$

2 $${{{\mathrm{10Ca}}}}^{ + 2} + 6{{{\mathrm{H}}}}_2{{{\mathrm{PO}}}}_4^ - + 2{{{\mathrm{OH}}}}^ - \to {{{\mathrm{Ca}}}}_{10}({{{\mathrm{PO}}}}4)_6({{{\mathrm{OH}}}})_2 + 12{{{\mathrm{H}}}}^ +$$

### Composite preparation and characterization

Afterward, PCL/DMF solution (12% W/V) was prepared at 60 °C; simultaneously, the HA suspension was prepared in DMF in various concentrations of HA. Then it was added to the PCL solution dropwise and stirred at 60 °C for 6 h. Then PCL/HA solution was dried for 72 h at room temperature and maintained in the desiccator. Table 1 demonstrates the detail of weights in composite preparation.

**Table 1:** Weight of PCL, HA, and their solvents for preparation of different composites

Composite	PCL (gr)	HA (gr)	PCL solvent (ml)	HA solvent (ml)
PCL95%HA5%	3	0.158	25	3.16
PCL90%HA10%	3	0.33	25	3.3
PCL85%HA15%	3	0.529	25	3.52
PCL80%HA20%	3	0.75	25	3.75

The X-Ray Diffraction (Equinox model, France) with Cu–Kα radiation was employed to identify the crystalline structure of PCL and HA. Scanning Electron Microscopy (Philips Company, Netherlands) was used to observe the nanocomposite structure and show the presence of HA particles in the polymeric matrix. Before the observation by SEM, the specimens were coated with gold by the coating device (Quorum technology, Emitech, SC7620, England).

### Filament fabrication and rheological characterization

The rheometer device (DAVENPORT model, US) was used for filament fabrication and evaluating rheological properties. With the aim of filament fabrication, the temperature for PCL and PCL/HA was set at ≈105 °C and ≈130 °C, respectively. It is worthy to note that the absence of a heat insulator is a possible reason for the lack of precise temperature in various situations. The material was heated for 15 min and compacted in a cylinder to become homogenous and reduction of undesirable air bubbles. Then, the force of 50 N was applied to the material to fabricate the filament. After that, the filaments were vacuum dried and kept in a desiccator before usage. The diameter of filaments was considered 1.75 mm for use in a standard FFF device.

To determine the flowability of the material during the printing process, the melt flow index (MFI) of them was measured. The process was conducted according to the ASTM-D1238 standard. The device was set at 180 °C (printing temperature), and the material was heated for 6 min. Then, 21.19 N force was applied to the piston in a cylinder. The amount of outlet material from the cylinder was separated after a minute. This process was done ten times, and the mass of the whole material was measured.

#### Mechanical properties of PCL and PCL/HA Filaments

The tensile strength device (SANTAM STM-1 model, Iran) was used to evaluate the tensile strength of the filaments. The maximum force applied to the samples and accuracy of the device were 800 N and 0.1 N, respectively. The tensile force was applied to the filaments at a 1 mm/min speed. The dimension of filaments for this test was 80 mm in height and an average diameter of 1.6 mm. The test was executed in 3 repetitions. The stress-strain curves of the specimens were obtained from recorded data. The yield stress and Young’s modulus of filaments were calculated from the stress-strain curve.

### Design and fabrication of scaffolds

**Fig. 2:**
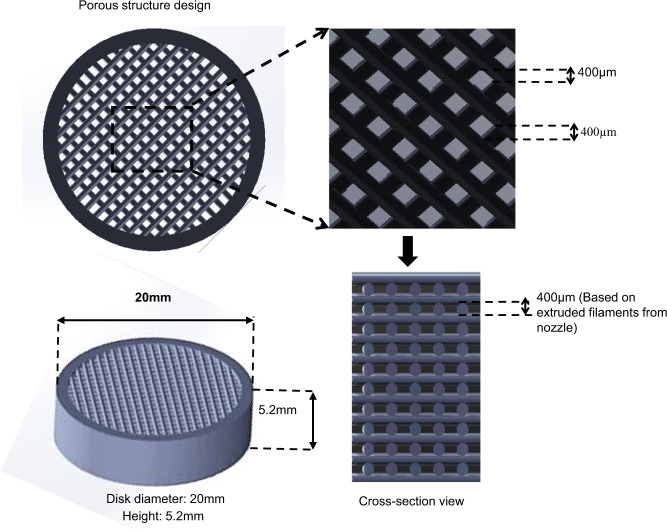
Design of three-dimensional (3D) scaffold in SolidWorks 2017 software. It shows over-view and cross-section view of scaffolds with diameter and height of 20 mm and 5.2 mm, respectively

The porous scaffold structure was designed using SolidWorks 2017 software. The disc shape was considered as an overall shape for all scaffolds with a pore size of 400 µm. As the FFF device has a nozzle size of 0.4 mm, the struts of the scaffold were designed with a diameter of 400 μm (Fig. 2). Indeed, the scaffolds were designed by considering bone scaffold properties (better regeneration) and the limitation of the device for printing scaffolds.

FFF 3D-printer (Generous3D, Persia Company, Iran) was employed to 3D-print the specimens. The nozzle and ambient temperatures were set at ≈180 °C and 25 ± 2 °C during the process, respectively (due to the absence of a heat insulator, maybe this temperature is not precise in other circumstances).

### Characterization of scaffolds

#### Macrostructure evaluation

Scanning Electron Microscopy (Philips Company, Netherlands) was used to observe the cross-section and over-view of scaffolds. Moreover, the formation of secondary HA crystals and cell adhesion on the scaffolds were observed using SEM. Before the observation by SEM, the specimens were coated with gold by the coating device (Quorum technology, Emitech, SC7620, England).

#### Mechanical properties evaluation

The tensile strength device (SANTAM STM-1 model, Iran) was used for the evaluation compression strength of scaffolds. The maximum force applied to the samples and accuracy of the device were 800 N and 0.1 N, respectively. The compression force was applied at a 1 mm/min speed to the scaffolds. The dimension of the scaffolds for doing the compression test was 6 mm diameter and 12 mm height. First, the scaffolds were printed with a diameter of 10 mm and the same height. Then they were punched to obtain the dimension mentioned above. The test was executed in three repetitions. The stress-strain curves of the specimens were obtained from recorded data. The Young’s modulus of scaffolds was calculated from stress-strain curves.

#### In vitro biological evaluation

To perform the cell evaluation, the specimens were sterilized by a gamma-ray device (Gama cell 220, Canada). The irradiation dose was adjusted to be 25 kGy. The extraction procedure was accomplished according to the ISO10993-5 protocol. Scaffolds were placed in culture media and maintained at 37 °C for 7 and 14 days to fulfill biocompatibility and ALP (For each 3 cm^2^ sample’s surface, 1 ml of culture media was added). Biological properties evaluation of PCL and PCL/HA scaffolds was performed using a human osteoblast cell line (MG-63, NCBI; National Cell Bank of Iran). RPMI (Gibco, USA) with 100 U·ml^−1^ penicillin, 10% (v/v) fetal bovine serum (FBS, Gibco, USA) and 100 g·ml^−1^ streptomycin was utilized to culture MG-63 cells in an incubator with a temperature of 37 °C, humidified atmosphere, and presence of 5% CO_2_ [25].

##### Biocompatibility

First, cells were cultured into a 96-well plate (Nunc, Denmark), including 100 µl media with 10^4^ cells (MG-63) per well. After 24 h, the culture medium was removed, and (10 µL FBS + 90 µL extract) was added. After 24 h, the solution was replaced with 100 µL of 0.5 M MTT solution and stayed in an incubator for 4 h. Then, the solution was removed, and isopropanol (100 µL, Sigma, US) was augmented per well for 30 min. In the end, an ELISA reader (STAT FAX 2100, US) was employed to read formazan crystals’ optical density (OD) at 570 nm. The cell viability was determined by normalizing the results compared to the control sample.

##### Cell adhesion

The culture media was removed to observe cell adhesion, and after washing with PBS, the scaffolds were fixed with 3.5% glutaraldehyde. After 2 h, the samples were washed with deionized water and ethanol (50, 60, 70, 80, and 96%). The presence of cells on the scaffolds was observed utilizing SEM.

##### Alkaline Phosphatase (ALP) Activity

The ALP activity was measured to determine the differentiation of the MG-63 cells on the scaffolds. A density of 2 × 10^4^ cells was seeded on the scaffold per well (12-well plate), then cells on each scaffold were incubated for 7 and 14 days in extract solution and 10% FBS [26]. After 7 and 14 days, culture media were collected. ALP assay kit and auto-analyzer (Hitachi 902, Japan) were used to measure the ALP activity.

##### Alizarin red Staining

Alizarin red staining was used to indicate the deposition of calcium ions around MG-63 cells. Primarily, 2 × 10^4^ MG-63 cells were incubated on samples in a 12-well plate for 10 days. The samples were washed three times with saline serum (0.9%) after removal from culture media. After that, the cells were fixed utilizing paraformaldehyde (2%) for 20 min and then stained with alizarin red staining solution (2%, pH 4.2) for 45 min at room temperature. The samples were washed with the saline serum before Optical Microscopic (OM) observation.

#### Bioactivity evaluation

The scaffolds were immersed in Simulated Body Fluid (SBF) prepared by the Kokubo method [27]. Then, they stayed in an incubator (37 °C) for 21 days. Afterward, the scaffolds were washed using deionized water and waited at room temperature to dry.

### Statistical analysis

The results were represented as the mean ± SD. Statistical analyses were accomplished utilizing one-way analysis of variance (ANOVA) and *p < 0.05, **p < 0.01, ***p < 0.001, and ****p < 0.0001 were demonstrated for significant difference between samples.

## Results and discussion

### Hydroxyapatite Characterization

**Fig. 3:**
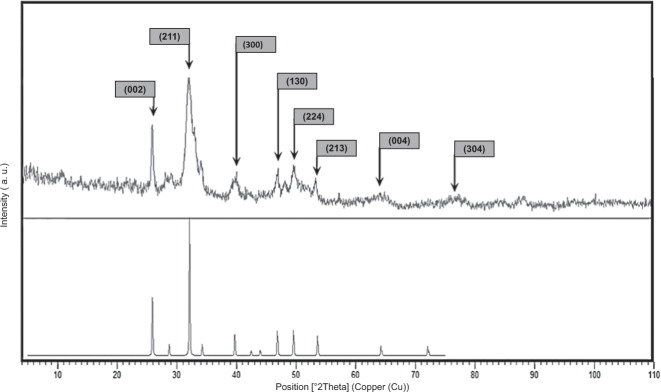
**A** XRD pattern of synthesized hydroxyapatite to show the crystalline structure of HA

The crystalline structure of HA is shown in Fig. 3 using the X-ray diffractometer. The peaks were according to the peaks in JCPDS cards. The index peaks of HA were observed at (002) and (211) [28]. The peaks were sharp and distinct, which showed that the HA powder was well-crystallized. According to Bragg’s law, the appearance of peaks in smaller angles leads to the shorter distance between the atomic planes of material. Scherer’s law expresses that the small width at Full Width at Half Maximum (FWHM) shows a smaller material crystal size.

In Eqs. (1) and (2), the parameters of n, D, d, λ, θ, β indicate positive integer, crystal size, atomic plane distance, X-ray wavelength, Bragg’s angle, line broadening at half the maximum intensity.

The parameters of h, k, l are the miller notation of the crystallographic plane (h, k, l); the miller notation of highest peak crystallographic plane, (211), and Eqs. (3–5) was utilized to determine the parameters in Table 2. According to this table, the synthesized HA particles are Nanoparticles with an HCP structure and crystal size of about 28.8 nm. Also, the distance between the crystallographic planes was obtained at about 2.79 Angstrom.

3 $$n\lambda = 2d\sin \theta$$

4 $$D = \frac{{0.9 \times \lambda }}{{\beta \,{\mathrm{cos}}\,\theta }}$$

5 $$c = l \ast d\quad a = d \ast \sqrt {\frac{4}{3} \ast \left( {h^2 + hk + k^2} \right)}$$

**Table 2:** Synthesized HA crystalline properties

	c (A^◦^)	a (A^◦^)	Maximum intensity (2θ)	Crystallite size(A^◦^)	Atomic plane distance(A^◦^)
**HA**	2.790	8.5236	32.05	288	2.790

### Composite films characterization and rheological behavior

Figure 3(A) shows SEM images of the composite films. Hydroxyapatite particles were shown as white particles in the polymeric matrix. By increasing the HA percentage, the presence of these particles has increased in the polymeric matrix and caused their agglomeration in some parts; the small surface area and higher surface energy of these particles affected their accumulation. Indeed, the fine powder is more likely to get agglomerated [29]. In other parts, well dispersion of hydroxyapatite particles was observed. It is vital to have a proper distribution of bioceramic reinforcement into the polymeric matrix to prevent stress concentration on one side, especially for bone applications. It also affects the mechanical properties of the scaffold. The presence of porosities in the composite films is probably related to the removal of solvent molecules.

The presence of HA particles, also crystalline phases of HA and PCL in PCL/HA10% and PCL/HA20% composite films were determined by X-Ray Diffraction (Fig. 3(B)). The HA peaks in PCL/HA composites and pure HA powder were placed similarly; however, the peaks were weak compared to the XRD pattern of HA powder due to the low crystallinity. The incorporation of PCL and HA has reduced the intensity of the peaks [5]. The particular peaks of PCL and HA were seen at the angles of 2θ = 23.67, 21.37° [30, 31], and 2θ = 32,27°, respectively. The spectra showed that with an increase of HA, the crystalline phases of PCL and HA have not changed. Figure 3(C) demonstrates the rheological behavior of PCL and PCL/HA (5%, 10%, 15%, and 20%).

**Fig. 4:**
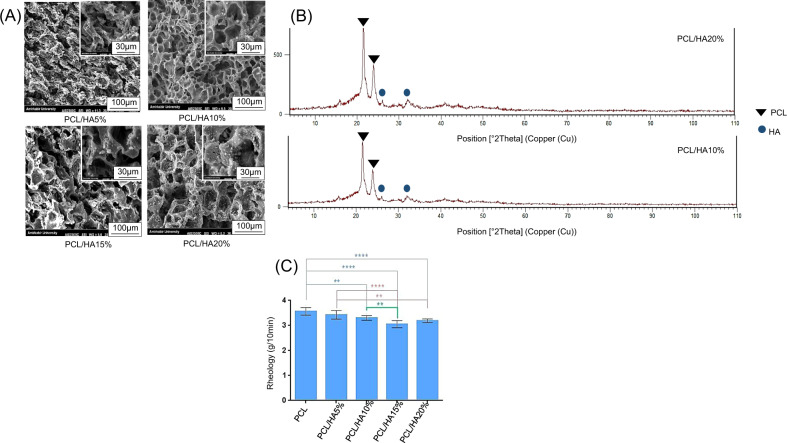
**A** SEM images of PCL/HA5%, PCL/HA10%, PCL/HA15% and PCL/HA20% composite films. White particle corresponds to the presence of HA. **B** XRD pattern of PCL/HA10% and PCL/HA20% composite films. The particular peaks of PCL and HA are at the angles of 2θ = 23.67, 21.37°, and 2θ = 32,27°, respectively. **C** Rheology behavior of PCL, PCL/HA5%, PCL/HA10%, PCL/HA15%, and PCL/HA20% composites at 180 °C. Error bars show standard deviation (*n* = 3). The rheology has decreased by increasing of HA percentage. (**p < 0.01, ****p < 0.0001)

The Melt Flow Index (MFI) of the molten polymer significantly affects the extrusion process [32]. The most flowability and the least viscosity are related to PCL. PCL/HA5% and PCL showed approximately the same behavior. Therefore, the addition of 5% HA has no significant effect on rheological behavior. The addition of HA particles has decreased the rheology; thus, the viscosity has increased. Indeed, the surface area and interaction between the polymeric matrix and inorganic particles will increase by adding bioceramic particles, resulting in higher viscosity and inherent friction [19, 33]. Hence, the higher percentage of HA leads to nonprintable filaments due to the lack of flowability Fig. 4.

### Mechanical Properties of PCL and PCL/HA Filaments

Filaments were fabricated utilizing the rheometer device. The diameter of the filaments must be in accordance with the characteristics of the FFF device. The PCL and PCL/HA filaments had a diameter of 1.75 ± 0.15 mm, which was usable in the 3D printer. During the process of filament fabrication, the uniform force was applied to the piston to obtain filament with a uniform diameter. Pre-heat and initial force to the material led to homogeneity and lack of bubbles.

**Fig. 5:**
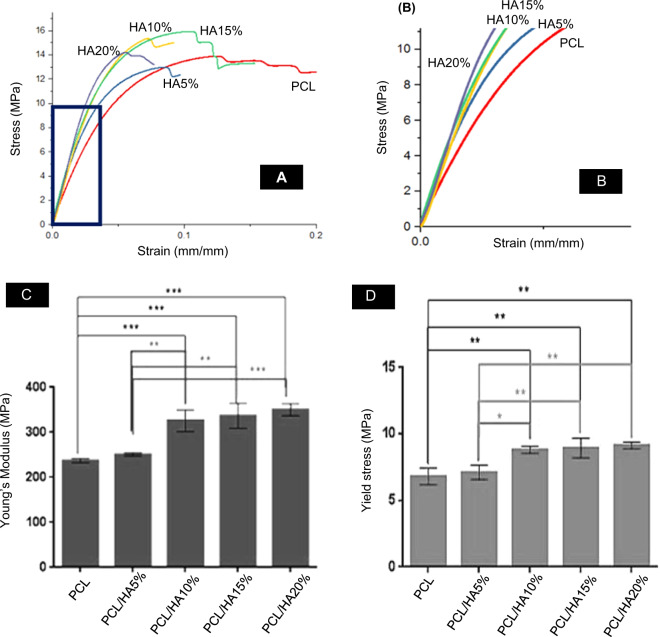
**A** Stress-strain curve of fabricated PCL, PCL/HA5%, PCL/HA10%, PCL/HA15%, and PCL/HA20% under tensile strength. **B** Elastic section of stress-strain curves of filaments under tensile strength. **C**, **D** Young’s modulus and yield stress of PCL, PCL/HA5%, PCL/HA10%, PCL/HA15%, and PCL/HA20% filaments under tensile strength which is calculated from the stress-strain curves. Error bar presents the standard deviation. (*p < 0.05, **p < 0.01, and ***p < 0.001, *n* = 3)

Figure 5 highlights the influence of HA inclusion on the mechanical properties of the PCL-based filaments. The steep slope could be interpreted as stiffer filaments. On the other hand, a low slope shows the lower tensile strength and easily deformed specimens. The elasticity modulus of specimens was calculated by the slope of the elastic area of the curves. The elasticity modulus of PCL, PCL/HA5%, PCL/HA10%, PCL/HA15%, and PCL/HA20% filaments is 237.27 ± 4.67, 250.82 ± 7.1 ± 0.52, 326.03 ± 24.27, 336.67 ± 27.18, and 340.72 ± 4.33 MPa, respectively. Moreover, yield stress was calculated by drawing the straight line at the same slope of 0.001 of each curve. The yield stress of PCL, PCL/HA5%, PCL/HA10%, PCL/HA15%, and PCL/HA20% is 6.80 ± 0.60, 7.1 ± 0.52, 8.8 ± 0.26, 8.29 ± 0.72, and 9.13 ± 0.25 MPa, respectively. The tensile strength of the filaments demonstrates the bulk properties of a material. Young’s modulus, which expresses the stiffness of the material, is calculated from the elastic region of stress-strain curves. Yield stress indicates the maximum tolerable stress by the material before entering the plastic region. In fact, at the yield stress, the linear behavior of the material is over, and the plastic deformation begins. Figure 5(C, D) shows Young’s modulus and yield stress diagrams. These figures show that PCL/HA10%, PCL/HA15% and PCL/HA20% (*P* < 0.001) has significant difference with PCL. The addition of a 5% hydroxyapatite had no significant effect on PCL properties. In all the samples, with an increase of HA, Young’s modulus and yield stress increased. According to the mixture rule, composite properties are the combination of the properties of the constituent materials. Therefore, adding HA particles to the PCL has increased the elastic deformation range of the material and prevented its entry into the permanent deformation region (plastic). Hydroxyapatite is an excellent bioceramic for hard tissue engineering due to its high similarity to bone structure; however, its brittleness is its main limitation. Therefore, a combination of hydroxyapatite particles into the polymeric matrix makes an appropriate material for bone application with suitable mechanical properties. Table 3 shows Young’s modulus and yield stress of all the filaments. According to this table, Young’s modulus and yield stress have increased by adding HA particles. The bulk properties of PCL/HA20% filament have increased by 30% compared to the PCL filament. It is expected that by adding 25% HA, the mechanical properties of filaments increase significantly, making the filament less flexible and unsuitable for printing.

**Table 3:** Young’s modulus and yield stress of tensile strength of filaments

Material	Young’s modulus (MPa)	Yield stress (MPa)
PCL	237.27 ± 4.67	6.80 ± 0.60
PCL/HA5%	250.82 ± 3.20	7.1 ± 0.52
PCL/HA10%	326.03 ± 24.27	8.8 ± 0.26
PCL/HA15%	336.67 ± 27.18	8.92 ± 0.72
PCL/HA20%	340.72 ± 4.33	9.13 ± 0.25

### Scaffold characterization

#### Macrostructure of Scaffolds

**Fig. 6:**
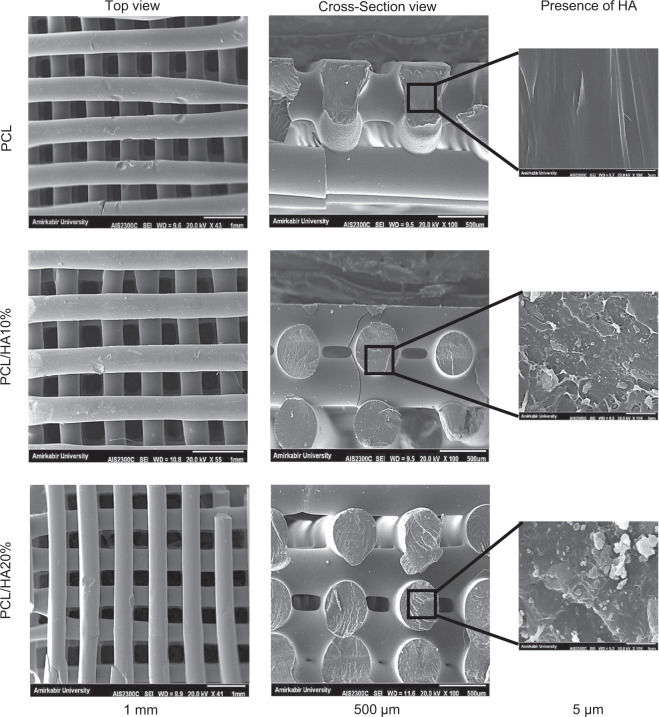
SEM images of PCL, PCL/HA10%, and PCL/HA20% scaffolds. The first and second columns show top and cross-section views of scaffolds (The scale bar is 1 mm and 500 µm, respectively). The third column pictures show the presence of HA particles in the polymeric matrix. (The scale bar is 5 µm)

PCL and PCL/HA porous scaffolds were successfully fabricated utilizing FFF. The uniformity of pore and deposited filament showed the ability of the FFF process to fabricate the scaffold according to the design. This process was accomplished by melting and extruding filaments through the nozzle in sequence layers. Figure 6 shows the SEM images of PCL, PCL/HA10%, and PCL/HA20% scaffolds from top and cross-section views, also HA dispersion in the polymeric matrix. PCL and PCL/HA strands were deposited well through the nozzle. The space between deposited strands in each scaffold layer is in the range of 400 ± 30 µm. Strands were printed regularly according to the design. PCL/HA10% and PCL/HA20% scaffolds had neater deposited struts than the PCL scaffold. The incorporation of HA into the PCL matrix influenced the solidification and resulted in less shrinkage. Therefore, more shrinkage has occurred in the PCL scaffold as it took more time to solidify. The temperature must be optimized to have a suitable junction between printed layers in the FFF printing process. Besides, the material should lose heat quickly to prevent flowability and dimension changes [15]. The cross-section images show strands forming each layer of the scaffolds. The strands had circle geometry without intense distortion, and they are parallel to each other in successive layers. The diameter of PCL/HA strands is closer to the initial design due to less shrinkage. The nozzle size was 400 µm, and the deposited filaments had a circular section with approximately 400 µm diameter. The images show the proper distribution of HA particles in PCL in the cross-section of scaffolds. In some parts, HA particles have been agglomerated [29].

#### Compression Strength of Scaffolds

**Fig. 7:**
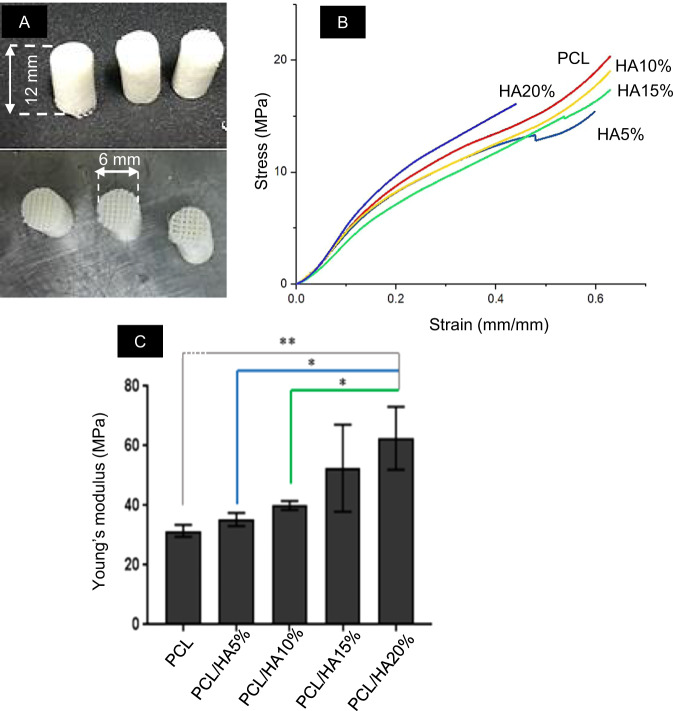
**A** Punched specimens for the mechanical testing. The diameter and height of the samples were 6 mm and 12 mm, respectively. **B** The stress-strain curves of PCL, PCL/HA5%, PCL/HA10%, PCL/HA15%, and PCL/HA20% scaffolds under compression test (*n* = 3). **C** The Young’s modulus of PCL, PCL/HA5%, PCL/HA10%, PCL/HA15%, and PCL/HA20% scaffolds under compression test. Error bar indicateس the standard deviation. (*p < 0.05 and **p < 0.01, *n* = 3)

The compression force is significantly essential among various forces applied to the bone. The compression test was performed to evaluate the impact of HA addition in 3D-printed scaffolds. Young’s modulus was obtained from the linear part (elastic region) of the curves. Figure 7 shows that Young’s modulus of PCL/HA scaffolds has increased tremendously. The Young’s modulus of scaffolds was obtained in the range of 30–70 MPa (Table 4), which is suitable for use in the cancellous bones as Young’s modulus of the cancellous bone is in the range of 20–500 MPa [6]. The addition of HA particles has enhanced the mechanical properties of scaffolds; therefore, PCL/HA scaffolds can withstand more compression force due to higher stiffness [11].

**Table 4:** Young’s modulus of compression test of scaffolds

Material	Young’s modulus (MPa)
PCL	31.522 ± 1.985
PCL/HA5%	35.306 ± 2.180
PCL/HA10%	40.025 ± 1.44
PCL/HA15%	52.527 ± 14.65
PCL/HA20%	62.67 ± 10.55

Compared to the recently reported PCL/HA scaffolds [23], our scaffolds showed better mechanical properties for bone tissue engineering applications. Indeed, hydroxyapatite makes the material more rigid and more resistant to plastic deformation; hence, by applying compression force, the PCL/HA collapses for a longer time, especially in a composition with a higher percentage of hydroxyapatite. The PCL/HA20% scaffold was significantly different from PCL, PCL/HA5%, and PCL/HA10%. Young’s modulus of PCL/HA20% has increased by 50% and 43% compared to PCL and PCL/HA5% scaffolds.

#### Biological Evaluation

**Fig. 8:**
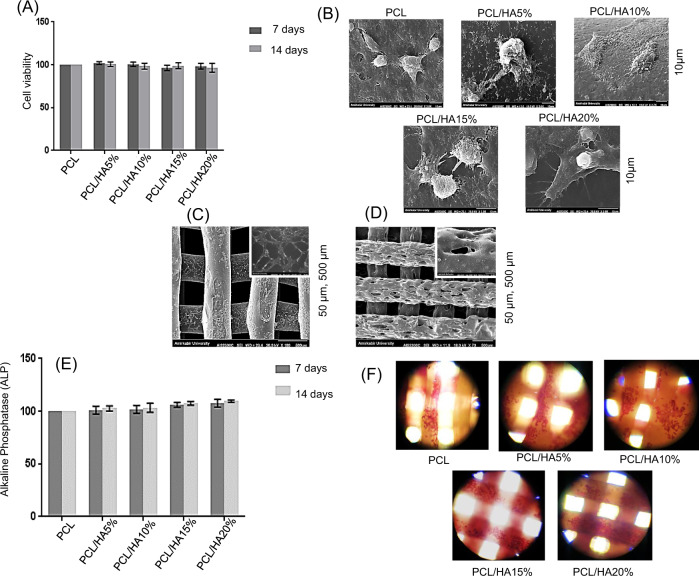
**A** Cell viability of MG-63 cell line after contact with PCL, PCL/HA5%, PCL/HA10%, PCL/HA15%, and PCL/HA20% scaffold’s extraction. The extraction time was performed for 7 and 14 days. **B** SEM images of cell adhesion on PCL, PCL/HA5%, PCL/HA10%, PCL/HA15% and PCL/HA20% after 3 days. **C**, **D** SEM image of MG-63 cell adhesion PCL/HA20% scaffolds after 3 ad 14 days. **E** Alkaline Phosphatase (ALP) activity of PCL, PCL/HA5%, PCL/HA10%, PCL/HA15%, and PCL/HA20% after 7 and 14 days. **F** Microscopic images of calcium deposition on PCL, PCL/HA5%, PCL/HA10%, PCL/HA15%, and PCL/HA20% scaffolds after 10 days. The red color shows the amount of deposited calcium. (The error bar shows the standard deviation, *n* = 3)

MTT assay was utilized to evaluate the cell viability on the scaffolds (Fig. 8A). The extraction of samples was accomplished for 7 and 14 days, and the extraction of the PCL scaffold was considered as a control. According to Fig. 8A, all the samples showed appropriate cell viability after 24 h contact with the extract, and it is expected that the addition of HA particles raises biocompatibility [11]. The PCL/HA scaffolds did not show any significant difference from the control sample in two periods of 7 and 14 days. Figure 8B shows cell adhesion on the PCL, PCL/HA5%, PCL/HA10%, PCL/HA15%, and PCL/HA20% scaffolds after three days. Cell adhesion on the scaffolds was observed using SEM. The cells are attached and placed separately on the surface; their shape is almost spherical, and they tend to spread on the scaffold. Figure 8(C, D) shows the adhesion of MG-63 cells after 3 and 14 days on PCL/HA20% scaffolds. According to SEM images, the boundary between the cells is clear after 3 days, and the cells are attached separately on the scaffold’s surface. However, the cell’s boundary has disappeared after 14 days, and the cellular monolayer has formed on the surface. Also, the cells are spreading over the pores on scaffolds (Fig. 8D).

The ALP enzyme is a marker for bone mineralization and differentiation. The expression of this enzyme was evaluated after 7 and 14 days (Fig. 8E). HA particles’ addition has increased the ALP activity after 7 and 14 days, and this increase is more evident after 14 days. Besides, the ALP expression has increased over time; therefore, the result confirms the cell proliferation and differentiation of MG-63 cells after 7 and 14 days. Thus, the material composition and time duration have impacted ALP activity.

Alizarin red staining is commonly utilized to paint the calcium deposition of the cells. Figure 8F shows pictures of all the scaffolds after alizarin red staining utilizing an Optical Microscope (OM). The red color corresponds to calcium deposition. Calcium deposition was observed in all the scaffolds; however, its amount varies. Calcium deposition is higher in PCL/HA%15% and PCL/HA20% scaffold. It seems that the presence of the HA particle has enhanced the Ca deposition.

#### Bioactivity

**Fig. 9:**
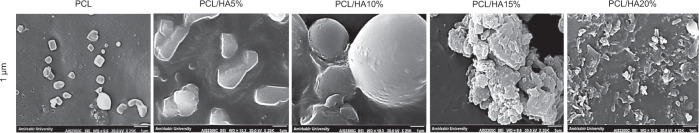
SEM images of PCL, PCL/HA5%, PCL/HA10%, PCL/HA15%, and PCL/HA20% scaffolds after 21 days of soaking in SBF show the bioactivity of scaffolds after this duration. (The scale bar for all the images is 1 µm)

The formation of apatite in vitro expresses the bioactivity of biomaterials in vivo [34]. Scanning Electron Microscopy (SEM) was utilized to observe scaffolds after 21 days of soaking in the SBF (Fig. 9). Apatite formed on the surface of all scaffolds. The amount of it is higher in PCL/HA15% and PCL/HA20% scaffolds. There are two main reasons for less apatite formation on PCL scaffold: bioinertness of PCL and dissolving a significant amount of this apatite in SBF over time [35].

The surface porosity affects apatite formation. Daljeet Singh et al. showed that the more the surface of a material has porosity, the more apatite forms on this surface. On the contrary, apatite formation is less on the surface with low porosity [36]. The surface of the deposited struts of each scaffold is smooth; therefore, it seems the FFF process has affected the apatite formation.

## Conclusion

In this study, predesigned PCL and PCL/HA scaffolds were fabricated successfully via FFF. The range of HA percentage was chosen based on the PCL/HA composition rheology and printability of the filaments. The addition of HA particles has decreased the rheology. The scaffolds represented good interconnectivity pores and mediocre HA dispersion across the scaffold. The addition of HA particles increased the mechanical properties of filaments and scaffolds. In the filaments, Young’s modulus and yield stress have increased. According to the mixture rule, composite properties are a combination of constituent properties. By adding 25% HA, filaments are expected to become less flexible and harder; however, a higher percentage of hydroxyapatite leads to brittle and non-usable filaments in the FFF device. The distribution of HA particles into PCL has probably increased the mechanical properties, especially Young’s modulus. For instance, Young’s modulus of PCL/HA20% has increased by 30% compared to PCL.

Moreover, the MTT assay was utilized to evaluate cell viability on the scaffolds. All the samples had good cytocompatibility, and it would be anticipated that addition of HA particles raise the biocompatibility in vivo. The cells are individually and shaped spherically on the surface. After 14 days, the boundary between the cells is no longer clear, and the cellular monolayer was formed on the scaffold. Alkaline phosphatase evaluation shows that the addition of HA caused higher ALP activity in the PCL/HA scaffold than PCL.

Furthermore, the amount of alizarin red in the PCL/HA specimens is higher than in control. In all samples, apatite was formed but in different amounts. More secondary apatite formation was observed in samples containing 15 and 20% hydroxyapatite. However, the bioinertness of PCL and the dissolving of a significant amount of this apatite in SBF over time are the reasons for less formation of secondary HA crystals on the PCL scaffold. In conclusion, these scaffolds showed that commercial and low-cost FFF device is promising technique for fabricating composite bone scaffolds.

## Abbreviations

3D: three dimensional
PCL: Polycaprolactone
HA: Hydroxyapatite
(FFF): Fused Filament Fabrication
SBF: Simulated Body Fluid
ALP: Alkaline Phosphatase

## References

[1] Donnaloja F (2020). Natural and synthetic polymers for bone scaffolds optimization. Polymers.

[2] Rodriguez G (2013). Influence of hydroxyapatite on extruded 3D scaffolds. Procedia Eng.

[3] Eshraghi S, Das S (2010). Mechanical and microstructural properties of polycaprolactone scaffolds with one-dimensional, two-dimensional, and three-dimensional orthogonally oriented porous architectures produced by selective laser sintering. Acta biomaterialia.

[4] Naghieh S (2016). Numerical investigation of the mechanical properties of the additive manufactured bone scaffolds fabricated by FDM: The effect of layer penetration and post-heating. J Mech Behav Biomed Mater.

[5] Park SA, Lee SH, Kim WD (2011). Fabrication of porous polycaprolactone/hydroxyapatite (PCL/HA) blend scaffolds using a 3D plotting system for bone tissue engineering. Bioprocess Biosyst Eng.

[6] Sabir MI, Xu X, Li L (2009). A review on biodegradable polymeric materials for bone tissue engineering applications. J Mater Sci.

[7] Bose S, Roy M, Bandyopadhyay A (2012). Recent advances in bone tissue engineering scaffolds. Trends Biotechnol.

[8] Loh QL, Choong C (2013). Three-dimensional scaffolds for tissue engineering applications: role of porosity and pore size. Tissue Eng Part B: Rev.

[9] Park SH (2012). Scaffolds for bone tissue engineering fabricated from two different materials by the rapid prototyping technique: PCL versus PLGA. J Mater Sci: Mater Med.

[10] Boschetto A, Bottini L (2014). Accuracy prediction in fused deposition modeling. Int J Adv Manuf Technol.

[11] Kim J-W (2017). Production of poly (ε-caprolactone)/hydroxyapatite composite scaffolds with a tailored macro/micro-porous structure, high mechanical properties, and excellent bioactivity. Materials.

[12] Mohamed OA, Masood SH, Bhowmik JL (2015). Optimization of fused deposition modeling process parameters: a review of current research and future prospects. Adv Manuf.

[13] Abdelaal OA, Darwish SM (2011). Fabrication of tissue engineering scaffolds using rapid prototyping techniques. World Academy of Science, Engineering and Technology. Int J Mech, Aerosp, Ind, Mechatron Manuf Eng.

[14] Koons GL, Diba M, Mikos AG (2020). Materials design for bone-tissue engineering. Nat Rev Mater.

[15] Mazzanti V, Malagutti L, Mollica F (2019). FDM 3D printing of polymers containing natural fillers: a review of their mechanical properties. Polymers.

[16] Bharadwaz A, Jayasuriya AC (2020). Recent trends in the application of widely used natural and synthetic polymer nanocomposites in bone tissue regeneration. Mater Sci Eng: C.

[17] Bruyas A (2018). Systematic characterization of 3D-printed PCL/β-TCP scaffolds for biomedical devices and bone tissue engineering: influence of composition and porosity. J Mater Res.

[18] Roh H-S (2017). Addition of MgO nanoparticles and plasma surface treatment of three-dimensional printed polycaprolactone/hydroxyapatite scaffolds for improving bone regeneration. Mater Sci Eng: C.

[19] Huang B, Bártolo PJ (2018). Rheological characterization of polymer/ceramic blends for 3D printing of bone scaffolds. Polym Test.

[20] Liu F (2018). Structural evolution of PCL during melt extrusion 3D printing. Macromol Mater Eng.

[21] Brunelli M, Perrault C, Lacroix D (2017). Mechanical response of 3D Insert® PCL to compression. J Mech Behav Biomed Mater.

[22] Huang B (2018). Polymer-ceramic composite scaffolds: the effect of hydroxyapatite and β-tri-calcium phosphate. Materials.

[23] Gatto ML (2021). Biomechanical performances of PCL/HA micro-and macro-porous lattice scaffolds fabricated via laser powder bed fusion for bone tissue engineering. Mater Sci Eng: C.

[24] Tahriri M, Solati-Hashjin M, Eslami H (2008). Synthesis and characterization of hydroxyapatite nanocrystals via chemical precipitation technique. Iran J Pharm Sci.

[25] Mehrjoo M (2015). Effect of magnesium substitution on structural and biological properties of synthetic hydroxyapatite powder. Mater Express.

[26] Jung S-C, Lee K, Kim B-H (2012). Biocompatibility of plasma polymerized sandblasted large grit and acid titanium surface. Thin Solid Films.

[27] Kokubo T, Takadama H (2006). How useful is SBF in predicting in vivo bone bioactivity?. Biomaterials.

[28] Murugan R, Ramakrishna S (2005). Crystallographic study of hydroxyapatite bioceramics derived from various sources. Cryst Growth Des.

[29] Šupová M (2009). Problem of hydroxyapatite dispersion in polymer matrices: a review. J Mater Sci: Mater Med.

[30] Fabbri P (2010). Porous scaffolds of polycaprolactone reinforced with in situ generated hydroxyapatite for bone tissue engineering. J Mater Sci: Mater Med.

[31] Shoja M (2015). Preparation and characterization of poly (ε-caprolactone)/Tio 2 micro-composites. Dig J Nanomaterials Biostructures.

[32] Barnes, HA, JF Hutton, and K Walters, *An introduction to rheology*. Vol. 3. 1989: Elsevier.

[33] Jiang W (2012). Morphology, wettability, and mechanical properties of polycaprolactone/hydroxyapatite composite scaffolds with interconnected pore structures fabricated by a mini‐deposition system. Polym Eng Sci.

[34] Kokubo, T, *Bioceramics and their clinical applications*. 2008: Elsevier.

[35] Hernandez I, Kumar A, Joddar B (2017). A bioactive hydrogel and 3D printed polycaprolactone system for bone tissue engineering. Gels.

[36] Singh D (2019). Synthesis, characterization, and bioactivity investigation of biomimetic biodegradable PLA scaffold fabricated by fused filament fabrication process. J Braz Soc Mech Sci Eng.

